# Benchmarking software tools for detecting and quantifying selection in evolve and resequencing studies

**DOI:** 10.1186/s13059-019-1770-8

**Published:** 2019-08-15

**Authors:** Christos Vlachos, Claire Burny, Marta Pelizzola, Rui Borges, Andreas Futschik, Robert Kofler, Christian Schlötterer

**Affiliations:** 1 Institut für Populationsgenetik, Vetmeduni Vienna, Veterinärplatz 1, Wien, 1210 Austria; 2 Vienna Graduate School of Population Genetics, Vienna, Austria; 3 Institute of Applied Statistics, Johannes Kepler University, Linz, 4040 Austria; 4 Plattform Bioinformatik und Biostatistik, Vetmeduni Vienna, Veterinärplatz 1, Wien, 1210 Austria

## Abstract

**Background:**

The combination of experimental evolution with whole-genome resequencing of pooled individuals, also called evolve and resequence (E&R) is a powerful approach to study the selection processes and to infer the architecture of adaptive variation. Given the large potential of this method, a range of software tools were developed to identify selected SNPs and to measure their selection coefficients.

**Results:**

In this benchmarking study, we compare 15 test statistics implemented in 10 software tools using three different scenarios. We demonstrate that the power of the methods differs among the scenarios, but some consistently outperform others. LRT-1, CLEAR, and the CMH test perform best despite LRT-1 and the CMH test not requiring time series data. CLEAR provides the most accurate estimates of selection coefficients.

**Conclusion:**

This benchmark study will not only facilitate the analysis of already existing data, but also affect the design of future data collections.

**Electronic supplementary material:**

The online version of this article (10.1186/s13059-019-1770-8) contains supplementary material, which is available to authorized users.

## Introduction

Experimental evolution is an extremely powerful approach to study adaptation in evolving populations [1, 2]. Apart from a well-controlled environment and a known demography, experimental evolution obtains much of its power from the use of replicated populations, which are evolving in parallel. The application of next-generation sequencing, called Evolve and Resequence (E&R) [3–5], allowed for genomic analyses of experimental evolution studies. Sequencing pools of individuals (Pool-Seq, [6]) has become the routine method to measure allele frequencies of entire populations across the whole genome. While the initial focus was on the comparison of allele frequencies between two groups, either two selection regimes or ancestral and evolved populations, the field is now recognizing the power of time series data to characterize the underlying evolutionary processes at unprecedented detail [7–10].

The great potential of E&R studies in combination with the continuously growing data sets of powerful experiments has driven the development of a diverse set of methods to detect selected SNPs, which change in allele frequency more than expected under neutrality [11–19]. Some of the published methods use this information to estimate the underlying selection coefficient and dominance [11, 14, 19, 20]. While publications reporting new software tools typically include some comparisons to previously published ones, a systematic comparison of the currently available tools with standardized data sets is still missing.

A major shortcoming of all comparisons of software tools for the detection of selection in E&R studies is that they are only targeted to evaluate the performance under the selective sweep regime [3, 21]. The underlying assumption of the selective sweep paradigm is that all loci are selected without any implicit or explicit connection to the phenotype. As a consequence, all loci that are not lost by genetic drift become ultimately fixed. Despite its central role in the molecular evolution literature, it is becoming increasingly clear that E&R studies need to consider phenotypes to understand the selection signatures. Many E&R studies use truncating selection where a defined phenotype is used to determine which individuals are contributing to the next generation [22–25]. The genomic signature of truncating selection is clearly distinct from selective sweeps [26]. Laboratory natural selection (LNS) is another widely used approach in E&R studies [2]. Rather than selecting for well-defined phenotypes, a polymorphic population is exposed to a novel environment and replicate populations evolve towards a new trait optimum. A characteristic property of this polygenic adaptation is genetic redundancy [7]. This implies different loci can contribute to the same phenotype in different replicates. As a consequence, not all loci show parallel selection signatures in all populations [27]. Because concordant behavior is an important feature for many software tools, it is not clear how well they perform with LNS and polygenic adaptation.

Here, we report the first benchmarking study, which evaluates the performance of software tools for the detection of selection in E&R studies for all three relevant scenarios: selective sweeps, truncating selection, and polygenic adaptation with a new trait optimum. Our benchmarking study includes software tools that use time series data, replicates, or only two time points. We show that the tools do not only differ dramatically in their computational time and inference accuracy, but we also demonstrate that depending on the underlying selection regime, the relative performance of the tools changes.

## Results and discussion

We evaluated the suitability of 10 different software tools with various underlying test statistics designed to identify the targets of selection in E&R studies. In total, the performance of 15 tests was evaluated for 3 different scenarios. Ten tests support multiple replicates whereas 5 are designed for a single replicate only. With the exception of the FIT2, CMH, LRT-1/2, and *χ*^2^ tests, all methods require time series data (for an overview of the evaluated tests, see Table 1; for a description of the tests, see the “Material and methods” section). Seven additional tools could not be evaluated due to technical difficulties (Additional file 1: Table S1).

**Table 1:** Overview of the evaluated tools

Tool	*t*	RAM	ts.	rep.	m/w	Description	Input	Output	lang.	Reference
*χ* ^2^	6 s	221 M	No	No	+/+	Pearson *χ*^2^ test for homogeneity (vectorized implementation)	freq, cov, Ne	p	R	[14]
E&R- *χ*^2^	8 s	306 M	Yes	No	+/+	*χ*^2^ test adapted to account for drift	freq, cov, Ne	p	R	[12]
CLEAR	3000 s	1100 M	Yes	Yes	+/+	Discrete HMM of allele trajectories under a WF model	sync,Ne	s, Ne, h, LL	Python	[11]
cmh	216 s	145 M	No	Yes	+/+	Test for homogeneity (similar to *χ*^2^) accounting for stratified data	sync	p	Perl/R	[13]
E&R-cmh	8 s	560 M	Yes	Yes	+/+	CMH test adapted to account for drift	freq, cov, Ne	p	R	[12]
LLS	1091 s (83 h)	340 M	Yes	Yes	+/+	Linear model with least square regression of logit-transformed allele frequencies	freq, cov, Ne	p, s, h	R	[14]
LRT-1	31 s	127 M	No	Yes	−/−	LRT of parallel selection	freq, cov, Ne	LRT, $\hat \delta $	Python	[15]
LRT-2	31 s	127 M	No	Yes	−/−	LRT of heterogeneous selection	freq, cov, Ne	LRT, *dx*_*r*_	Python	[15]
GLM	220 s	300 M	Yes	Yes	+/+	Quasibinomial GLM with replicates and time as predictors	freq	p	R	[16]
LM	157 s	300 M	Yes	Yes	+/+	LM with replicates and time as predictors	freq	p	R	[16]
BBGP	37 h	15 M	Yes	Yes	+/+	A Bayesian model of allele trajectories following a Gaussian process	sync	BF	R	[17]
FIT1	16 s	220 M	Yes	No	−/−	A *t* test with allele trajectories modeled as a Brownian process	freq	p	R	[18]
FIT2	68 s	220 M	No	Yes	−/−	A *t* test with allele frequencies differences between two time points	freq	p	R	[18]
WFABC	42 h	8 MB	Yes	No	+/+	ABC of WF dynamics with selection	freq, Ne (h)	BF, s	C++	[20]
slattice	41 h	250 M	Yes	No	+/+	HMM of allele trajectories under a WF model using an EM algorithm	freq, Ne (h)	s, LL	R	[19]

For each tool, we show the time required to analyze a small data set (*t*, either in seconds (*s*) or hours (*h*)), the memory requirements (RAM), if time series data may be used (ts.), if replicates are accepted (rep), if a manual and a walk-through is available (*m*/*w*), a short description, the required input, the generated output, the programming language (lang.), and the reference for LLS the time required to estimate the selection coefficient and the p-value (in brackets) is provided. *sync* file, *freq* allele frequency, *cov* coverage, *Ne* effective population size, *h* heterozygous effect, *p* value, *s* selection coefficient, *LRT* likelihood ratio test, *BF* Bayes factor, *LL* log-likelihood, $\hat \delta $ shared allele frequency change, *dx*_*r*_ change in allele frequency in a single replicate *r*

**Fig. 1:**
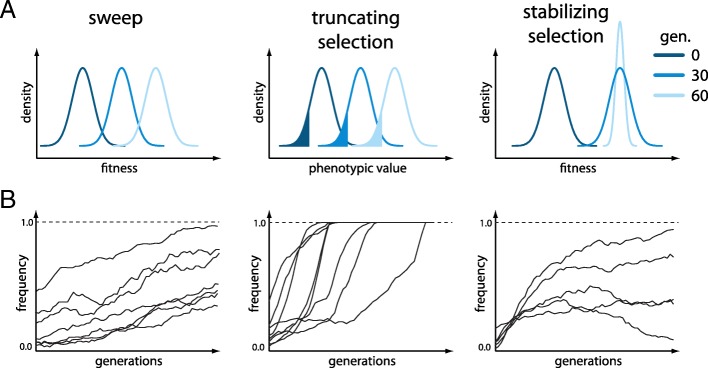
Overview of the simulated scenarios. **a** Response to selection with either fitness (sweep, stabilizing selection) or the phenotypic value (truncating selection) being displayed for three time points. For truncating selection, the fraction of culled individuals is indicated in color. With stabilizing selection, once the trait optimum is reached, selection acts to reduce the fitness variance within a population. **b** Schematic representation of the trajectories of the targets of selection expected for the three different scenarios

We simulated E&R studies under 3 different scenarios: selective sweeps, truncating selection, and stabilizing selection. Ten replicates of diploid populations each with 1000 individuals evolved for 60 generations, matching a powerful E&R design [21]. The founder population consisted of 1000 haploid chromosomes that capture the polymorphisms found on chromosome 2L of a natural *Drosophila melanogaster* population (Additional file 1: Figure S1; [28]). We used the *D. melanogaster* recombination maps [29], and regions with low recombination were excluded [21] (Additional file 1: Figure S1). Thirty targets of selection were randomly selected from all segregating sites with a frequency between 5 and 95% (Additional file 1: Figure S2). While we assumed a single selection coefficient of *s*=0.05 (Fig. 1, left panels) for the sweep model, for truncating selection, the effect size of the QTNs was drawn from a gamma distribution (shape=0.42 and scale=1) with a heritability of *h*^2^=1.0, and 20% of the individuals with the least pronounced phenotypes were culled (Fig. 1, middle panels). The effect size of the QTNs and the heritability for stabilizing selection were identical to truncating selection (shape=0.42,scale=1,*h*^2^=1.0), but additionally, a fitness function was specified such that the trait optimum was reached around generation 30–40. After the trait optimum is reached, stabilizing selection reduces phenotypic variation within a population (Fig. 1, right panels; Additional file 1: Figure S3). The three different scenarios typically result in different trajectories of selected alleles. The sweep architecture is characterized by selected loci that slowly rise in frequency and rarely get fixed until generation 50. For a quantitative trait architecture, truncating selection results in a rapid frequency increase of contributing alleles, often becoming fixed during the experiment. Different phases can be distinguished for stabilizing selection [27]. Initially, alleles rise in frequency, but when the populations approach the trait optimum, the contributing alleles experience a heterogeneous behavior in different replicates (Fig. 1; Additional file 1: Figures S4, S5, S6). Because these different trajectories could have important implications on the performance of the different software tools, we studied all three scenarios.

We evaluated the performance of each test with receiver operating characteristic (ROC) curves [30], which relate true-positive rate (TPR) to false-positive rate (FPR). A ROC curve having a TPR of 1.0 with a FPR of 0.0 indicates the best possible performance. Since the focus of E&R studies is the identification and characterization of selected alleles, we do not report the full ROC but used a small FPR threshold of 0.01 and computed the area under the partial ROC curve $\left (\text {pAUC}=\int _{0}^{0.01}f_{\text {ROC}}df\right)$ to assess the performance of a tool. With tools supporting the time series data, the allele counts at every tenth generation were used whereas the start and the end of the experiment were considered for tools not supporting the time series data. For tools not supporting multiple replicates, we restrict our analysis to the first of the 10 replicates. For each scenario, the performance was assessed by 100 different sets of randomly drawn targets of selection (random position and effect size) (Additional file 1: Figure S2) and the averaged ROC curves are displayed.

Whole-genome analyses evaluating the frequency changes of millions of SNPs can be computationally challenging, and the choice of software tools is also affected by CPU and memory requirements. We evaluated the speed and the memory requirements of the different approaches with a small data set (2 MB; sweep architecture; Additional file 1: Figure S1) on a powerful desktop computer (32 GB RAM; 2 × 2.66 GHz 6-Core Intel Xeon). For all tools, memory was not a limiting factor. The required RAM ranged from 8 to 1100 MB, which is readily met by standard desktop computers. Even more pronounced differences were observed for the time required to analyze 80,000 SNPs. The fastest tool, *χ*^2^ test, only required 6 s while the slowest tool, LLS, required 83 h (Table 1). Analyzing an E&R study of *D. melanogaster* with such a slow tool may require up to 192 days [assuming 4.5 million SNPs [7]]. We anticipate that the high computational demand of some tests may impose a severe burden for many users, even when species with a moderate genome size are being analyzed. Also for our benchmarking study, extensive computational demands posed a problem as each tool is evaluated with 300 data sets (3 scenarios and 100 sets of selected SNPs). To enable benchmarking all tools, we evaluated the performance of the slow tools (BBGP, LLS, and WFABC; Table 1) with a subset of the data (Additional file 1: Figure S1).

**Fig. 2:**
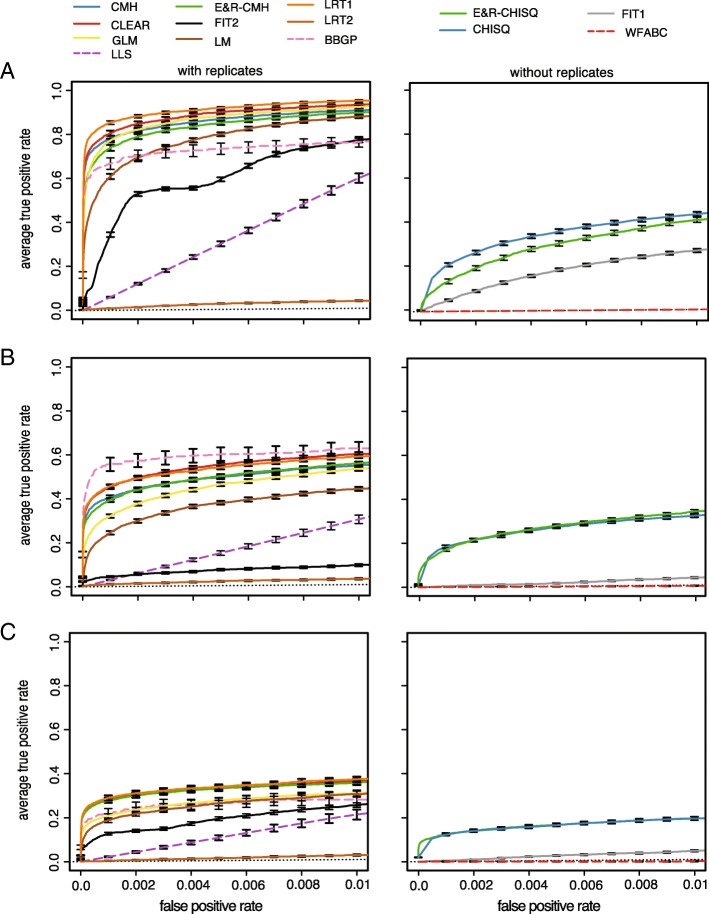
Performance of the tools under three different scenarios. The performance of tools supporting replicates (left panels) and not supporting replicates (right panels) was analyzed separately. For fast tools, the entire data set was analyzed (solid line) whereas a subset of the data was used for slow tools (dashed lines); The performance of a random classifier is shown as the reference (black dotted line). **a** Selective sweeps. **b** Truncating selection. **c** Stabilizing selection

For all scenarios, the software tools have a significantly different performance (Kruskal-Wallis test on pAUC values; with replicates *p*_sweep_<2.2×10^−16^,*p*_trunc_<2.2×10^−16^,*p*_stab_<2.2×10^−16^; without replicates *p*_sweep_<2.2×10^−16^,*p*_trunc_<2.2×10^−16^*p*_stab_<2.2×10^−16^; Fig. 2). Consistent with previous results [14], we found that tools using all 10 replicates generally outperform tools using only a single data set (Wilcoxon rank sum test with pAUC; best tool with 10 replicates vs. best tool without replicates; *p*_sweep_<2.2×10^−16^,*p*_trunc_=6.4×10^−14^,*p*_stab_<2.2×10^−16^).

### Selective sweeps

For selective sweeps, LRT-1 performed best among the tools supporting replicates (Wilcoxon rank sum test with pAUC; LRT-1 vs. CLEAR; *p*=4.7×10^−15^; Fig. 2) whereas the *χ*^2^ test had the best performance of tools not supporting replicates (Wilcoxon rank sum test with pAUC; *χ*^2^ vs. E&R- *χ*^2^; *p*<2.2×10^−16^); the low performance of LRT-2 was expected as this test was designed to identify replicate-specific response to selection [15]. Analyzing the subset of the data for all tools (not just the slower ones) does not affect the relative performance of the tools (Additional file 1: Figure S7). Interestingly, out of the three tools with the best performance, two tools do not require time series data (LRT-1, CMH test; Fig. 2).

### Truncating selection

The BBGP test was the best tool supporting replicates when truncating selection is used (Wilcoxon rank sum test with pAUC; BBGP vs. CLEAR; *p*=0.05; BBGP vs. LRT-1; *p*=0.03; (Fig. 2b). However, when the subset of the data was analyzed for all tools, the performance of BBGP was slightly worse than the performance of LRT-1 and CLEAR. We reason that this performance difference is the result of a similar performance of the best tools combined with a higher sampling variance when only a subset of the data is analyzed.

The performance of BBGP was better for truncating selection than for selective sweeps (Additional file 1: Figure S7). With truncating selection, selected loci quickly rise in frequency and the trajectories have the highest parallelism among the three scenarios, prerequisites for a good performance of BBGP (Carolin Kosiol, personal communication). This makes truncating selection the best scenario for the BBGP test. Interestingly, the performance of FIT1 and FIT2 was much worse with truncating selection than for selective sweeps. The rapid fixation of selected alleles before the end of the E&R experiment may be a problem for some tests. In agreement with this, we noticed that adding a small Gaussian random number to allele frequency estimates dramatically improved the performance of FIT2 (Additional file 1: Figure S8).

Of the tools not supporting replicates, the *χ*^2^ test and the E&R- *χ*^2^ test had the best performance (Wilcoxon rank sum test with pAUC; E&R- *χ*^2^ test vs. *χ*^2^ test; *p*=0.194; E&R- *χ*^2^ test vs. FIT1; *p*<2.2×10^−16^; Fig.2). Although these methods cannot be directly applied to multiple replicates, the *p* values obtained from single replicates could be combined using, for example, Fisher’s combination test [31] or the harmonic mean method [32].

### Stabilizing selection

Stabilizing selection is the most challenging scenario for all tools (Fig. 2). This is expected since selected alleles show a less pronounced allele frequency change with stabilizing selection and a more heterogeneous response in the different replicates (Fig. 1; Additional file 1: Figures S6, S9). Among the tests supporting multiple replicates, CLEAR, LRT-1, CMH, and E&R-CMH were the most powerful ones (first significant difference LRT-1 vs. GLM; Wilcoxon rank sum test with pAUC *p*=0.0001). The *χ*^2^ and E&R- *χ*^2^ again had the best performance of tools not supporting replicates (first significant difference *χ*^2^ vs. FIT1 (Wilcoxon rank sum test with pAUC *p*<2.2×10^−16^). Surprisingly, LRT-2, which was designed to identify replicate-specific allele frequency changes, still showed a weak performance although we found the most heterogeneous response to selection under this architecture (Additional file 1: Figure S9). This may either be due to the inherent difficulty of identifying a replicate-specific response to selection (replication provides important cues for distinguishing between genetic drift and selection) or that the heterogeneity among replicates is not pronounced enough (Additional file 1: Figure S9).

### Accuracy of estimated selection coefficients

**Fig. 3:**
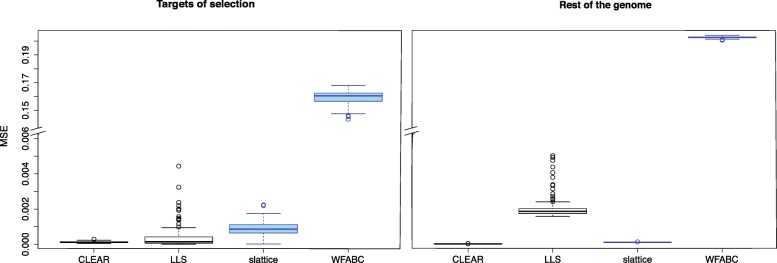
Accuracy of estimated selection coefficients in mean squared error (MSE). Results are shown for tests supporting (black) and not supporting (blue) multiple replicates

Four of the software tools estimate selection coefficients for the targets of selection (Table 1). We were interested in which of these methods estimates the selection coefficients most accurately. To address this question, we relied on the data from the selective sweep scenario for which the true selection coefficient of selected (*s*=0.05) and neutral (*s*=0.0) loci is known. We assessed the accuracy of the estimated selection coefficients by a sample-based estimate of the mean square error (*E*[(true−estimated)^2^]. Tools that support multiple replicates estimate selection coefficients more accurately than tools not supporting replicates (Wilcoxon rank sum test CLEAR vs. slattice; *p*_sel._<2.2×10^−16^,*p*_n.sel._<2.2×10^−16^; Fig. 3). CLEAR provided the most accurate estimates of the selection coefficients for both selected and neutral loci (Wilcoxon rank sum test with MSE; CLEAR vs. LLS; *p*_sel._=0.0016,*p*_n.sel._<2.2×10^−16^ Fig. 3). LLS provides fairly accurate estimates for selected loci but has a high error for neutral loci. LLS should therefore only be used on candidate loci for which sufficient statistical evidence for being selection targets has been established. slattice performs well with selected and neutral loci.

### Performance with experimental data

**Fig. 4:**
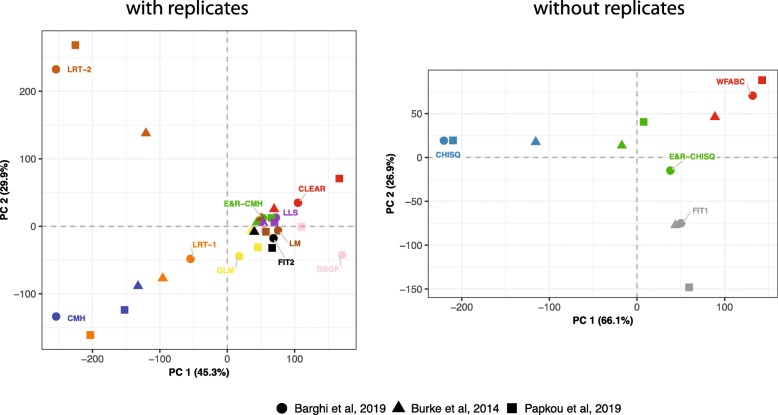
The tools perform similarly with data from different real E&R studies. We performed a PCA with the normalized test statistics for tools supporting (left panel) and not supporting replicates (right panel). Data are from E&R studies in *D. simulans* [7], *C. elegans* [33], and yeast [9]

Finally, we evaluated the performance of the tools with data from real E&R studies. We aimed to cover a wide range of organisms with different gene densities, recombination rates, and polymorphism pattern: (i) Barghi et al. [7] studied the adaptation of *Drosophila simulans* populations to hot conditions, (ii) Papkou et al. [33] investigated the co-adaptation of *Caenorhabditis elegans* to the pathogen *Bacillus thuringiensis*, and (iii) Burke et al. [9] studied the genomic response of *Saccharomyces cerevisiae* populations to laboratory conditions. Unlike computer simulations, the true targets of selection are not known for real data, which requires an alternative strategy to evaluate the performance of different tools. Here, we evaluate the tools by comparing the similarity of their performance for real data and compare this to the similarity for simulated data. We computed the pairwise correlation of the test statistics for all three real data sets (Additional file 1: Figure S10) and performed a Mantel test [34], which estimates the correlation among the distance matrices using permutations. Our results show that the tools have a similar performance with different real data sets (Mantel test, 10.000 permutations; multiple replicates *p*_Dsim−Cele_=9×10^−4^,*p*_Dsim−Scer_=5.5×10^−3^,*p*_Cele−Scer_=9.9×10^−5^; single replicate *p*_Dsim−Cele_=0.083,*p*_Dsim−Scer_=0.082,*p*_Cele−Scer_=0.080). A principal component analysis (PCA) based on the normalized test statistics also supports the similar performance of the tools with real data sets (Fig. 4). Finally, we found that the performance of the tools with real data is very similar to the performance with simulated data (Mantel test with average distance matrix; 10.000 permutations; multiple replicates *p*_real−sim_=5.2×10^−3^, single replicate *p*_real−sim_=0.085). We conclude that the evaluated tools show a very consistent behavior among a wide range of different real and simulated data.

## Conclusions

Across all evaluated scenarios, LRT-1, CLEAR, CMH, and E&R-CMH tests provided the most reliable identification of targets of selection in E&R studies. The best tool, LRT-1, is reasonably fast and can be readily used with genome-wide data. CLEAR, on the other hand, is computationally more demanding but additionally provides highly accurate estimates of selection coefficients, which also makes it a very promising tool. Whereas the classical CMH test requires simulations to obtain proper *p* value cutoffs for rejection; the E&R-CMH test provides adjusted *p* values that take drift and (if needed) also pooled sequencing into account.

Interestingly, out of the top performing tools, the LRT-1 and the CMH test do not require time series data. Therefore, with the evaluated test statistics, time series data are thus currently not required to maximize the power to identify the targets of selection. This is important, given that generating time series data comes at considerable costs, in our example about 3.5 × as high as for two time points. Time series data will however be important if accurate estimates of selection coefficients are required.

The parameters of the scenario of a polygenic trait evolving to a new optimum, which is reached after 30–40 generations, resulted in relatively parallel selection responses across replicates. Fewer selection targets, smaller population sizes, and more generations are expected to increase the heterogeneity among replicates. Further simulations are needed to evaluate how the different software tools are performing in cases of higher heterogeneity among replicates. Some evidence that this could affect the relative performance of the tools comes from BBGP, which performs much better with strong selection and highly parallel responses.

Finally, we made all files (simulation results, input for ROC curves, scripts, parameters) available on SourceForge https://sourceforge.net/p/erbenchmark, which allows researchers to compare the performance of novel test to the ones evaluated in this work.

This benchmarking study demonstrates that for different E&R scenarios, powerful software tools are available to detect selection targets. We anticipate that the community will greatly benefit from this first power evaluation across all three different scenarios, in particular as we have identified tools that perform uniformly very well across the three different scenarios. Our analyses also demonstrate that the comparison of two time points is very powerful and provides a cost-effective experimental design in combination with analyses that are also computationally cheap.

## Material and methods

### Evaluated tools

*χ*^2^ test. Pearson’s *χ*^2^ test for homogeneity relies on a 2 ×2 contingency table to compare for each SNP the allele counts from two different time points.

E&R *χ*^2^ test. A modification of the Pearson’s *χ*^2^ test which takes E&R-specific components of variance, in particular drift and pooled sequencing, into account [12].

Cochran-Mantel-Haenszel (CMH) test. The Cochran-Mantel-Haenszel (CMH) test [35] is a modified *χ*^2^ test (see above) that considers 2 ×2×*R* contingency tables, where *R* is the number of replicates. Similar to the *χ*^2^ test, the null hypothesis of the CMH test is that allele counts among samples are equal.

E&R-CMH test. A modified version of the CMH test [12] which takes E&R-specific components of variance, i.e., drift and pooled sequencing, into account. Pooled sequencing is modeled as binomial sampling.

Linear least squares (LLS). LSS implements a linear model on the logit-transformed allele frequency trajectories [14]. Population parameters such as *s* (and *h*) are estimated by least squares utilizing the consensus trajectories over multiple replicates. Deviations from neutrality are identified by comparison to neutral simulations.

Likelihood ratio test (LRT)-1. The LRT-1 test has been constructed to identify a parallel response to selection across multiple replicates, accounting for sampling noise [36]. Allele frequency differences between two time points are arcsine transformed [37] and assumed to be normally distributed with zero (neutral model) or non-zero (parallel model) mean. The test statistic is the likelihood ratio between the parallel and the neutral model.

Likelihood ratio test (LRT)-2. Following the approach taken with LRT-1, the LRT-2 test does not consider a shared response but uses an alternative hypothesis that permits for a replicate specific response to selection (heterogeneous model) [15]. The test statistics is the likelihood ratio between the heterogeneous and the neutral model.

LRT-1 and LRT-2 can be used at either window or SNP level; for the sake of consistency with other software tools, we only evaluated them SNP-based.

Generalized linear model (GLM). Allele frequencies are modeled using a generalized linear model [38] with a quasi-binomial error distribution, where *p* values are obtained from a Wald test to assess the time effect [16].

Linear model (LM). Allele frequencies are modeled as a linear model with a Gaussian error, and *p* values are obtained via *t* test. Time points and replicates are predictor variables [16].

Beta-binomial Gaussian process (BBGP). BBGP employs a beta-binomial Gaussian process to detect significant allele frequency changes over time [17]. The beta-binomial model corrects for the uncertainty arising from finite sequencing depth. This is a Bayesian method that does not provide *p* values but estimates Bayes factors (BFs) as a measure of evidence against neutrality.

Frequency increment test (FIT1). FIT1 uses a *t* test to test whether the expected allele frequency differences between two time points are significantly different from 0 [18].

Frequency increment test (FIT2). FIT2 works similarly to FIT1 but can use allele frequency data from several replicate populations [18].

Wright-Fisher approximate Bayesian computation (WFABC). WFABC estimates the effective population size, selection coefficients, and dominance ratio [20] using Wright-Fisher simulations and approximate Bayesian computation (ABC).

slattice. slattice provides a maximum likelihood estimator of *s* based on a hidden Markov model of allele frequency changes using the expectation-maximization algorithm [19, 39]. Furthermore, joint estimates of migration rate and spatially varying selection coefficients may be obtained at the single replicate level.

Composition of likelihoods for evolve and resequence experiments (CLEAR). To detect selected loci, CLEAR uses a hidden Markov model consisting of an underlying Wright-Fisher process and observed allele frequency counts from pool-sequenced organisms [11]. Besides estimating the selection coefficients, CLEAR also provides estimates for *N*_*e*_ and *h*.

### Simulations

We evaluated the performance of the software tools with individual-based forward simulations with MimicrEE2 [40]. The simulation parameters were chosen to match *D. melanogaster*, the most frequently used organism in E&R studies of an obligatory sexual organism (Table 2). The founder population consists of 1000 diploid individuals with haplotypes matching the polymorphism patterns of a natural *D. melanogaster* population [28]. For computational efficiency, we restricted our simulations to chromosome arm 2L (Additional file 1: Figure S1). We used the recombination estimates from Comeron et al. [29], and low recombining regions were excluded from the analysis as they inflate the noise [21]. In total, three different scenarios were simulated: a classic selective sweep model (selective sweeps), and two quantitative models, where the population evolved either under truncating or stabilizing selection (Fig. 1). For the classic sweep model, all selected loci had the same selection coefficient of *s*=0.05. For the quantitative models, the effect sizes of the QTNs were drawn from a gamma distribution with shape=0.42 and scale=1. The frequency of the selection targets ranged from 5 to 95%. For truncating selection, we selected the 80% of the individuals with the largest phenotypic values. This regime has a high power to identify the targets of selection [26, 41]. For stabilizing selection, we first estimated the mean and standard deviation of the phenotypes in the base population and then used a trait optimum that was shifted two standard deviations to the right of the population mean. With this selection regime, the trait optimum was usually reached around generation 40. This simulation setup allows for heterogeneity among replicates, since we expect that different SNPs will increase in frequency in the last 20 generations. We expect that this simulation setup will reduce the power to detect selected SNPs. Our aim was to show how the power of each test is affected by a given scenario and whether some tests perform equally well, independent of the simulated scenario.

**Table 2:** Overview of the default parameters used for the simulations

Parameter	Default value
Chromosome	2L
Population size (*N*)	1000
Number of causative loci	30
Number of generations	60
Replicates	10
Heritability	1.0
Recombination map	Comeron et al. [29]
Repetitions	100 (using different sets of selected SNPs)

### Details on benchmarking

We evaluated the performance of 15 different tests. Most tests were downloaded from the dedicated webpage, 2 were provided by the author and 2 were adapted to our data (Additional file 1: Table S2). If not mentioned otherwise, we used default parameters for each tool. For each site, we rescaled the allele counts to a uniform coverage of 100. To avoid numerical problems encountered by some methods with SNPs reaching an absorbing state (i.e., fixation or loss), we subtracted (added) a pseudocount of 1 to fixed (lost) SNPs.

For all tools requiring information about the effective population size, we provided the same estimate obtained separately for each simulation run. We provided the frequencies of random subsets of 1000 SNPs to estimate *N*_*e*_ with the poolSeq::estimateNe function [version 0.3.2; method =“P.planI”, truncAF =0.05, Ncensus = 1000; all other arguments set to default [14]]. We used the median of 100 trials with different random sets of SNPs. An independent estimate of *N*_*e*_ was obtained for each replicate. For tools requiring estimates of the dominance, we provided *h*=0.5. For CLEAR, we used a sync file as input.

Some tools provide estimates of *p* values or selection coefficients that are not compatible with downstream analysis (e.g., ROCR [42]). To nevertheless enable benchmarking these tools, we converted missing (NA) estimates of *p* values to 1.0, “infinite” estimates for negative log-transformed *p* values to 1,000,000, and “NA” estimates for selection coefficients to 0. The performance of each tool was assessed with receiver operating characteristic (ROC) curves [30], which relate the true-positive (TPR) to the false-positive rates (FPR). The TPR can be calculated as TP/(TP+FN) where TP stands for true positives and FN for false negatives. The FPR can be calculated as FP/(TN+FP), where FP refers to false positives and TN to true negatives. ROC curves and estimates of the area under the curve (AUC) were generated with ROCR [version 1.0-7; [42]]. Each ROC curve is the average over 100 replicates using different sets of selected SNPs. The ROC curve of WFABC under truncating selection is based solely on 29 different sets of selected SNPs as WFABC is extremely slow under this scenario. All files used in this work are available on SourceForge https://sourceforge.net/p/erbenchmark.

### Benchmarking with real data

We also evaluated the performance of the tools with data from three real E&R studies. Barghi et al. [7] allowed 10 *D. simulans* populations to adapt to hot conditions for 60 generations. The populations were sequenced each tenth generation. We used the 265,961 SNPs found in chromosome arm 2L. Papkou et al. [33] studied the co-adaptation of 6 replicated populations of *Caenorhabditis elegans* to the pathogen *Bacillus thuringiensis*. The populations were sequenced at generations 1, 12, and 22. We analyzed all 251,270 SNPs from the 5 autosomes. Burke et al. [9] studied the laboratory domestication in replicated *Saccharomyces cerevisiae* populations. The populations were sequenced at generations 0, 180, 360, and 540. We analyzed all 75,410 SNPs from the 12 chromosomes. As suggested by Iranmehr et al. [11], we solely investigated the replicates with consistent site frequency spectra over time (3, 7, 8, 9, 10, 11, 12).

We compared the performance of the tools with these data sets by computing the pairwise correlation (Spearman’s *ρ*) among the test statistics. We focused on the top 5% of the loci (union among all tools) as several tools yield identical test statistics for all non-significant loci. This could lead to low correlations among tools, mostly due to the non-significant SNPs. We converted the correlation matrices into a distance matrix ($\sqrt {(2(1-\rho))}$ [43]) and compared these matrices using the Mantel test [34] implemented in the ade4 R package [44]. PCA was performed with the scaled test statistics using the prcomp R function. PCA plots derived from the different data sets were superimposed using the Procrustes rotation [45, 46].

## Additional files

Additional file 1 Figures S1–S10 and Tables S1–S2. (PDF 759 kb)

Additional file 2 Review history. (DOCX 17 kb)

## Data Availability

The *D. simulans* data set is available at “PRJEB29281” [7], the *C. elegans* data set is available at “PRJNA475030” [33], the *S. cerevisiae* data set is available at “PRJNA265387” [9], and the source code is available under the GPL-3.0 license at SourceForge “https://sourceforge.net/projects/erbenchmark/” [47]. The version of the code used in the manuscript is available on Zenodo with DOI “10.5281/zenodo.3342556.”

## References

[1] Kawecki TJ, Lenski RE, Ebert D, Hollis B, Olivieri I, Whitlock MC (2012). Experimental evolution. Trends Ecol Evol.

[2] Garland T, Rose MR (2009). Experimental evolution: concepts, methods, and applications of selection experiments.

[3] Schlötterer C, Kofler R, Versace E, Tobler R, Franssen S (2015). Combining experimental evolution with next-generation sequencing: a powerful tool to study adaptation from standing genetic variation. Heredity.

[4] Long A, Liti G, Luptak A, Tenaillon O (2015). Elucidating the molecular architecture of adaptation via evolve and resequence experiments. Nat Rev Genet.

[5] Turner TL, D. A, Andrew S, Fields T, Rice WR, Tarone AM (2011). Population-based resequencing of experimentally evolved populations reveals the genetic basis of body size variation in *Drosophila melanogaster*. PLoS Genet.

[6] Schlötterer C, Tobler R, Kofler R, Nolte V (2014). Sequencing pools of individuals-mining genome-wide polymorphism data without big funding. Nat Rev Genet.

[7] Barghi N, Nolte V, Taus T, Jakšić AM, Tobler R, Mallard F, Dolezal M, Schlötterer C, Kofler R, Otte KA (2019). Genetic redundancy fuels polygenic adaptation in *Drosophila*. PLoS Biol.

[8] Lang GI, Rice DP, Hickman MJ, Sodergren E, Weinstock GM, Botstein D, Desai MM (2013). Pervasive genetic hitchhiking and clonal interference in forty evolving yeast populations. Nature.

[9] Burke M, Liti G, Long AD (2014). Standing genetic variation drives repeatable experimental evolution in outcrossing populations of Saccharomyces cerevisiae. Mol Biol Evol.

[10] Seabra SG, Fragata I, Antunes MA, Faria GS, Santos MA, Sousa VC, Simoes P, Matos M (2017). Different genomic changes underlie adaptive evolution in populations of contrasting history. Mol Biol Evol.

[11] Iranmehr A, Akbari A, Schlötterer C, Bafna V (2017). Clear: Composition of likelihoods for evolve and resequence experiments. Genetics.

[12] Spitzer K, Pelizzola M, Futschik A. Modifying the chi-square and the CMH test for population genetic inference: adapting to over-dispersion. 2019. http://arxiv.org/abs/1902.08127. Accessed 30 July 2019.

[13] Kofler R, Pandey RV, Schlötterer C (2011). PoPoolation2: identifying differentiation between populations using sequencing of pooled DNA samples (Pool-Seq). Bioinformatics.

[14] Taus T, Futschik A, Schlötterer C (2017). Quantifying selection with pool-seq time series data. Mol Biol Evol.

[15] Kelly JK, Hughes KA (2019). Pervasive linked selection and intermediate-frequency alleles are implicated in an evolve-and-resequencing experiment of Drosophila simulans. Genetics.

[16] Wiberg RAW, Gaggiotti OE, Morrissey MB, Ritchie MG (2017). Identifying consistent allele frequency differences in studies of stratified populations. Methods Ecol Evol.

[17] Topa H, Jónás Á, Kofler R, Kosiol C, Honkela A (2015). Gaussian process test for high-throughput sequencing time series: application to experimental evolution. Bioinformatics.

[18] Feder AF, Kryazhimskiy S, Plotkin JB (2014). Identifying signatures of selection in genetic time series. Genetics.

[19] Mathieson I, McVean G (2013). Estimating selection coefficients in spatially structured populations from time series data of allele frequencies. Genetics.

[20] Foll M, Shim H, Jensen JD (2015). Wfabc: a wright–fisher abc-based approach for inferring effective population sizes and selection coefficients from time-sampled data. Mol Ecol Resour.

[21] Kofler R, Schlötterer C (2014). A guide for the design of Evolve and Resequencing Studies,. Mol Biol Evol.

[22] Turner TL, Miller PM (2012). Investigating natural variation in *Drosophila* courtship song by the Evolve and Resequence approach. Genetics.

[23] Hardy C, Burke M, Everett L, Han M, Lantz K, Gibbs A (2017). Genome-wide analysis of starvation-selected *Drosophila melanogaster*—a genetic model of obesity. Mol Biol Evol.

[24] Griffin PC, Hangartner SB, Fournier-Level A, Hoffmann AA (2017). Genomic trajectories to desiccation resistance: convergence and divergence among replicate selected *Drosophila* Lines. Genetics.

[25] Castro J, Yancoskie MN, Marchini M, Belohlavy S, Beluch WH, Naumann R, Skuplik I, Cobb J, Nick H, Rolian C, Chan YF (2019). An integrative genomic analysis of the Longshanks selection experiment for longer limbs in mice. elife.

[26] Kessner D, Novembre J (2015). Power analysis of artificial selection experiments using efficient whole genome simulation of quantitative traits. Genetics.

[27] Franssen S, Kofler R, Schlötterer C (2017). Uncovering the genetic signature of quantitative trait evolution with replicated time series data. Heredity.

[28] Bastide H, Betancourt A, Nolte V, Tobler R, Stöbe P, Futschik A, Schlötterer C (2013). A genome-wide, fine-scale map of natural pigmentation variation in *Drosophila melanogaster*. PLoS Genet.

[29] Comeron JM, Ratnappan R, Bailin S (2012). The many landscapes of recombination in *Drosophila melanogaster*. PLoS Genet.

[30] Hastie T, Tibshirani R, Friedman J (2009). The elements of statistical learning: data mining, inference, and prediction.

[31] Edwards AWF (2005). RA Fischer, statistical methods for research workers, (1925). Landmark Writings in Western Mathematics 1640-1940.

[32] Wilson DJ (2019). The harmonic mean *p*-value for combining dependent tests. Proc Natl Acad Sci.

[33] Papkou A, Guzella T, Yang W, Koepper S, Pees B, Schalkowski R, Barg M-C, Rosenstiel PC, Teotónio H., Schulenburg H (2019). The genomic basis of red queen dynamics during rapid reciprocal host–pathogen coevolution. Proc Natl Acad Sci.

[34] Mantel N (1967). The detection of disease clustering and a generalized regression approach. Cancer Res.

[35] Agresti A (2002). Categorical data analysis.

[36] Kelly JK, Koseva B, Mojica JP (2013). The genomic signal of partial sweeps in *Mimulus guttatus*. Genome Biol Evol.

[37] Sokal RR, Rohlf FJ (1995). Biometry.

[38] McCullagh P. Generalized linear models: Routledge; 2018.

[39] Dempster AP, Laird NM, Rubin DB (1977). Maximum likelihood from incomplete data via the em algorithm. J R Stat Soc Ser B (Methodol).

[40] Vlachos C, Kofler R (2018). MimicrEE2: genome-wide forward simulations of Evolve and Resequencing studies,. PLoS Comput Biol.

[41] Vlachos C, Kofler R (2018). Optimizing the power to identify the genetic basis of complex traits with Evolve and Resequence studies. PLoS Comp Biol.

[42] Sing T, Sander O, Beerenwinkel N, Lengauer T (2005). ROCR: visualizing classifier performance in R. Bioinformatics.

[43] Revelle W (2018). Psych: procedures for psychological, psychometric, and personality research.

[44] Dray S, Dufour A-B (2007). The ade4 package: implementing the duality diagram for ecologists. J Stat Softw.

[45] Gower JC (1975). Generalized procrustes analysis. Psychometrika.

[46] Oksanen J, Blanchet FG, Friendly M, Kindt R, Legendre P, McGlinn D, Minchin PR, O’Hara RB, Simpson GL, Solymos P, Stevens MHH, Szoecs E, Wagner H. Vegan: Community Ecology Package. 2018. R package version 2.5-2. https://CRAN.R-project.org/package=vegan. Accessed 30 July 2019.

[47] Vlachos C, Burny C, Pelizzola M, Borges R, Futschik A, Kofler R, Schlötterer C. Benchmarking software tools for detecting and quantifying selection in evolve and resequencing studies. Souce code. SourceForge https://sourceforge.net/projects/erbenchmark/. 10.1186/s13059-019-1770-8 PMC6694636 31416462

[48] Terhorst J, Schlötterer C, Song YS (2015). Multi-locus analysis of genomic time series data from experimental evolution. PLoS Genet.

