# Supplementary material for: Benchmarking software tools for detecting and quantifying selection in evolve and resequencing studies
Source: Genome Biol. 2019 Aug 15;20:169. doi: 10.1186/s13059-019-1770-8 (PMC6694636; doi:10.1186/s13059-019-1770-8)
Supplement: Supplementary file 1 — Figures S1–S10 and Tables S1–S2. (PDF 759 kb) [file 13059_2019_1770_MOESM1_ESM.pdf]

Supplement to "Benchmarking software tools for detecting and quantifying selection in Evolve and Resequencing studies"

July 19, 2019

Supplementary figures

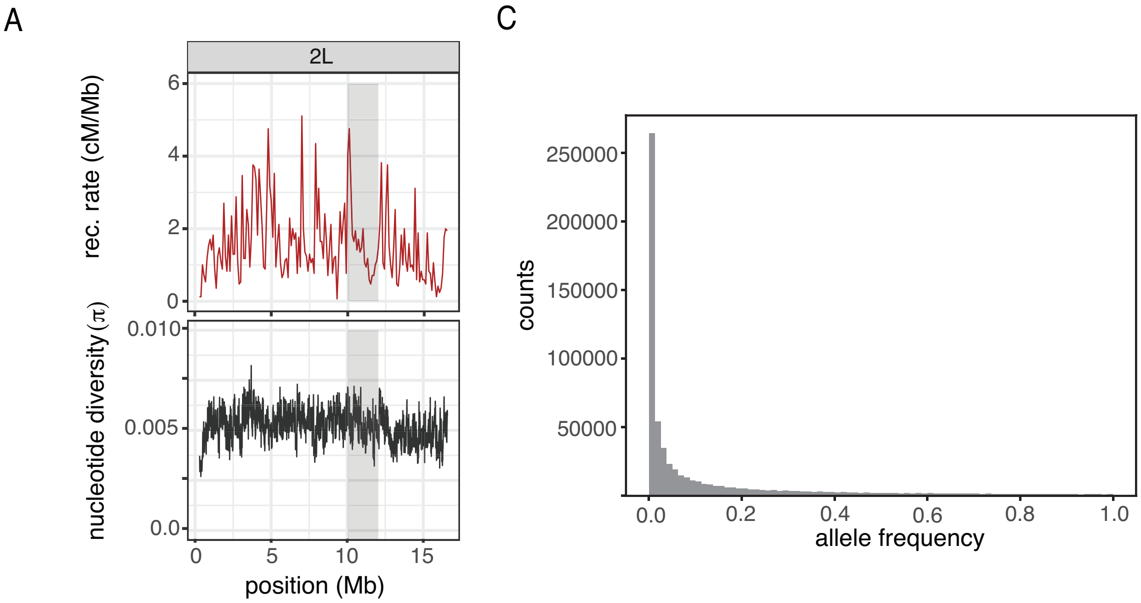

Fig. S1: Overview of the simulated base population. We aimed to capture the genomic landscape of chromosome arm 2L of *D. melanogaster*. A) The recombination rate (top panel; from Comeron et al. (2012)) and the nucleotide diversity (bottom panel) of the base population. The nucleotide diversity mirrors the level of polymorphism of a natural *D. melanogaster* population caught in Vienna (Bastide et al., 2013). The window size size is 100kb. The shaded area shows the genomic subset used for slow tools ($10 - 12$Mbp). C) Site frequency spectrum of derived alleles.

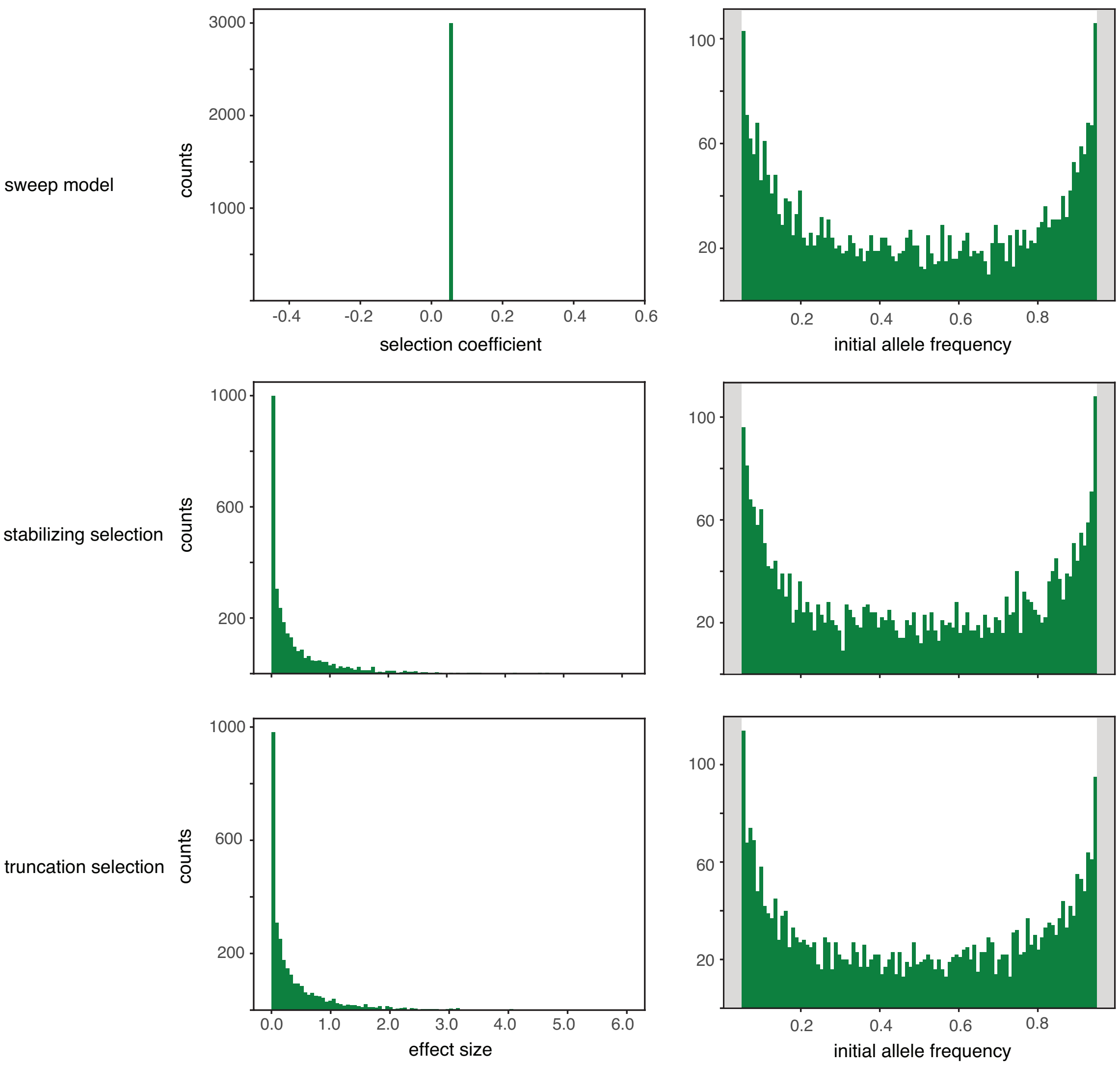

Fig. S2: Overview of the effect sizes (left) and the starting allele frequency of the targets of selection for the three simulated scenarios. The sum over all 100 replicates is shown ($3,000$ loci; $30 * 100$). For the sweep model all selected loci had an identical effect size whereas for the quantitative models (stabilizing and truncating selection) the effect sizes were drawn from a gamma distribution with $shape = 0.42$ and $scale = 1$. Note that all selected loci had an initial allele frequency between 5% and 95% (grey shades indicate excluded regions).

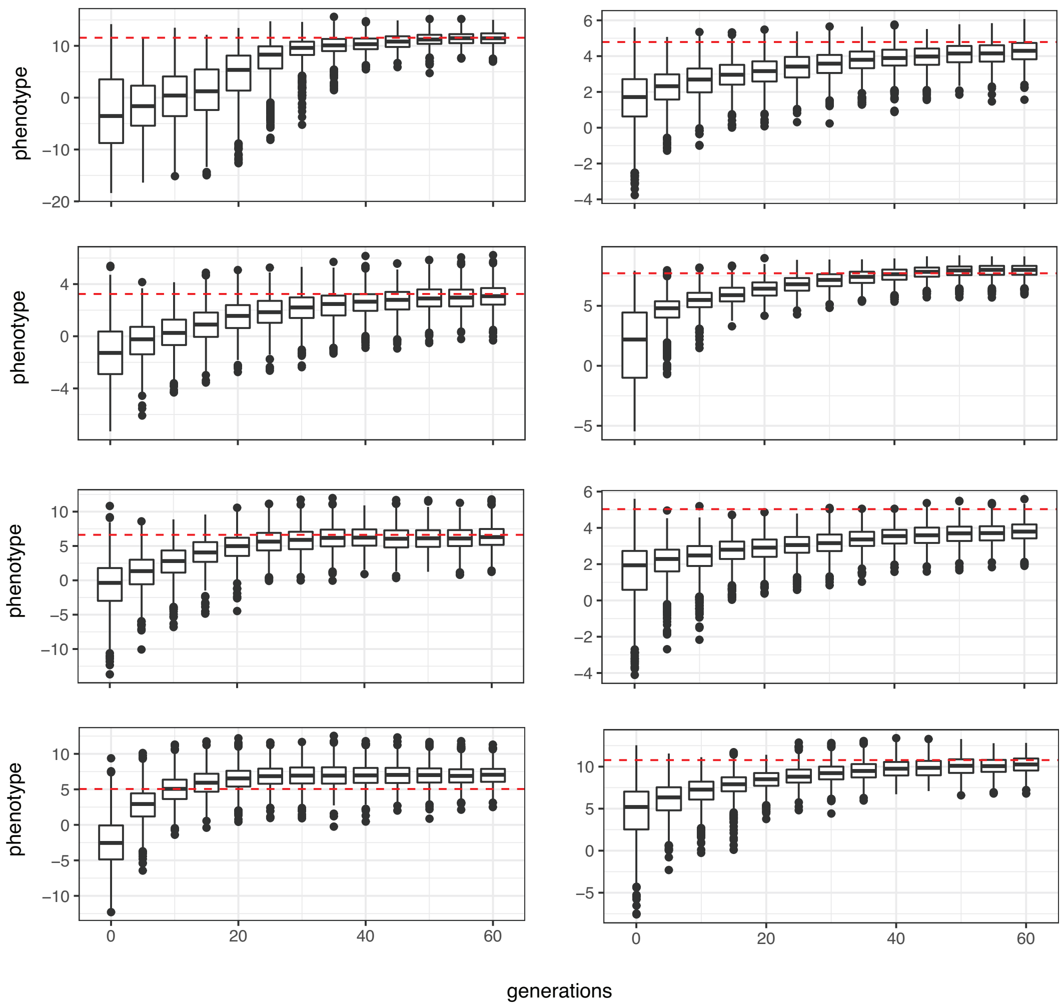

Fig. S3: Boxplots showing phenotypic values for individuals of a population at different generations during an E&R studies with stabilizing selection. Red dashed lines indicate the trait optimum. Eight randomly drawn replicates are shown (out of 1000 simulated ones).

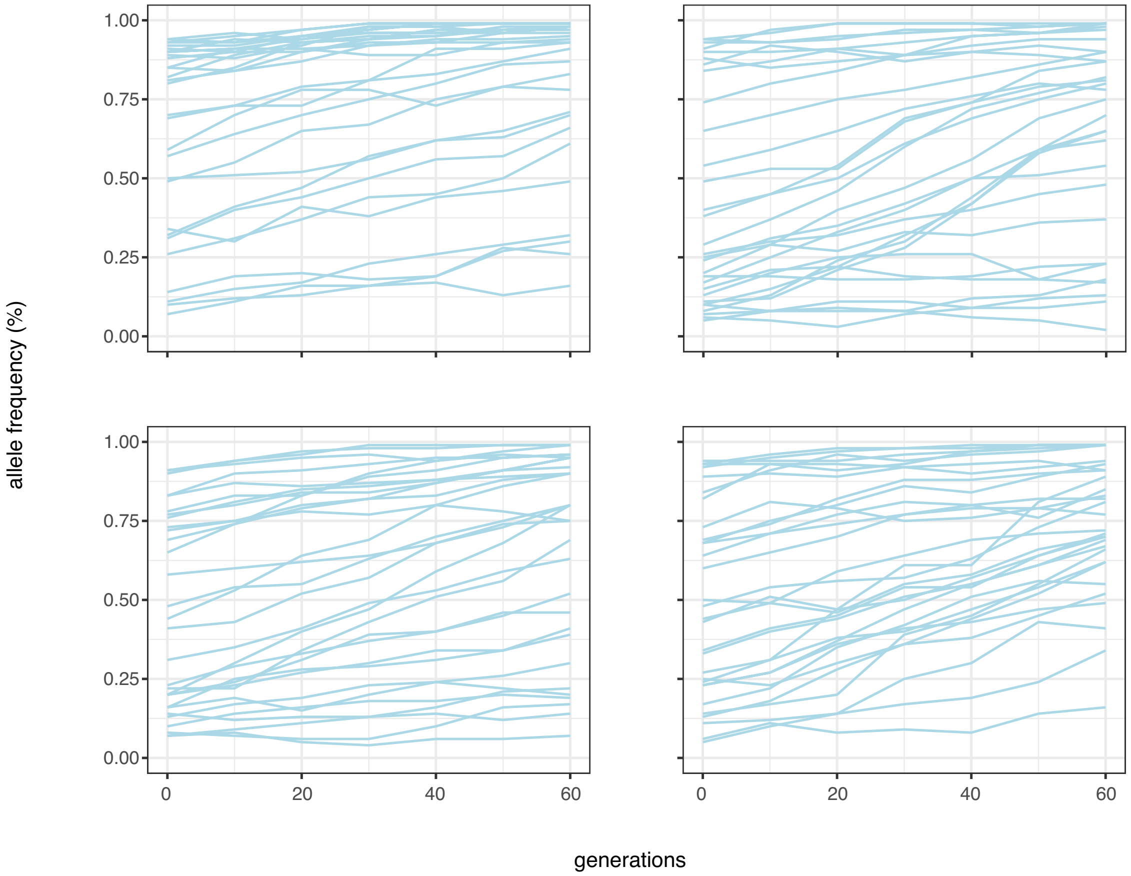

Fig. S4: Trajectories of selected SNPs for the sweep model. Four randomly drawn replicates are shown (out of 1000 simulated ones).

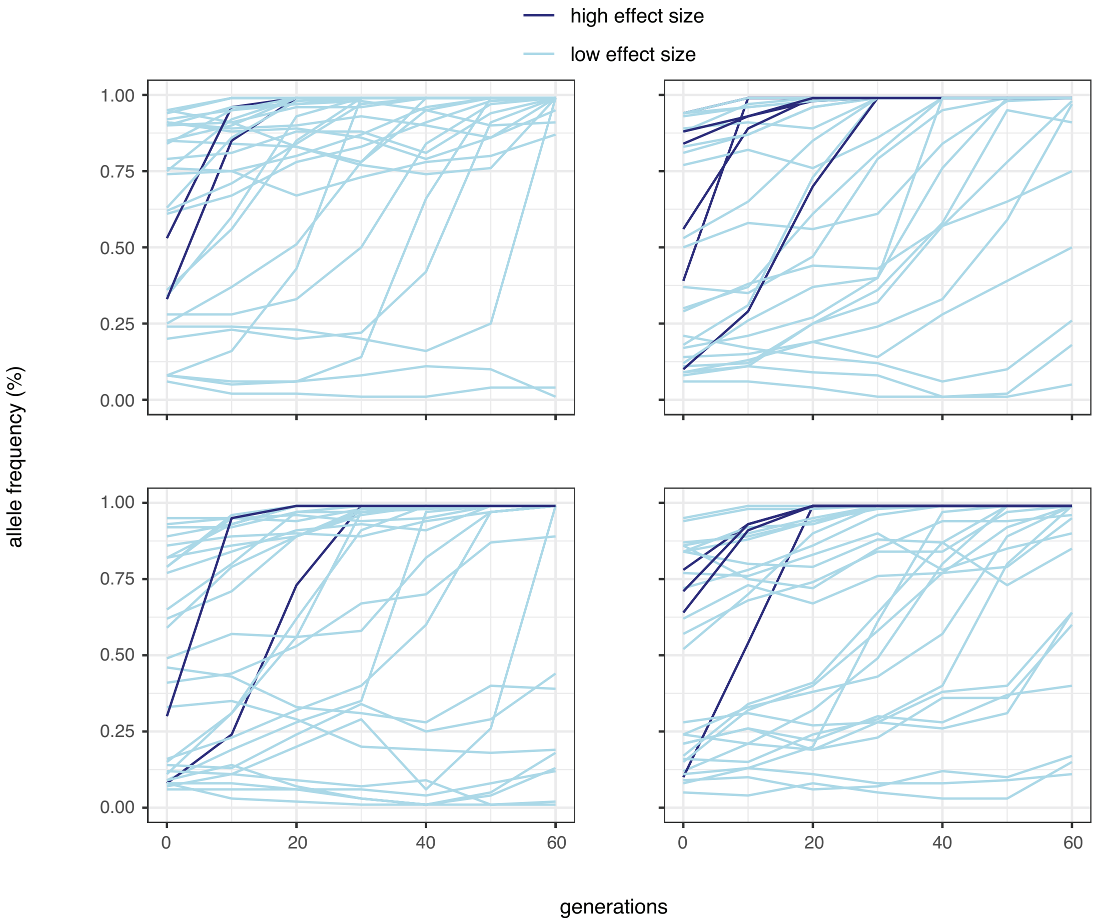

Fig. S5: Trajectories of the quantitative trait loci for truncating selection. Trajectories are shown for strong (dark blue; $e > 1$) and weak effect loci (light blue; $e \leq 1$). Four randomly drawn replicates are shown (out of 1000 simulated ones).

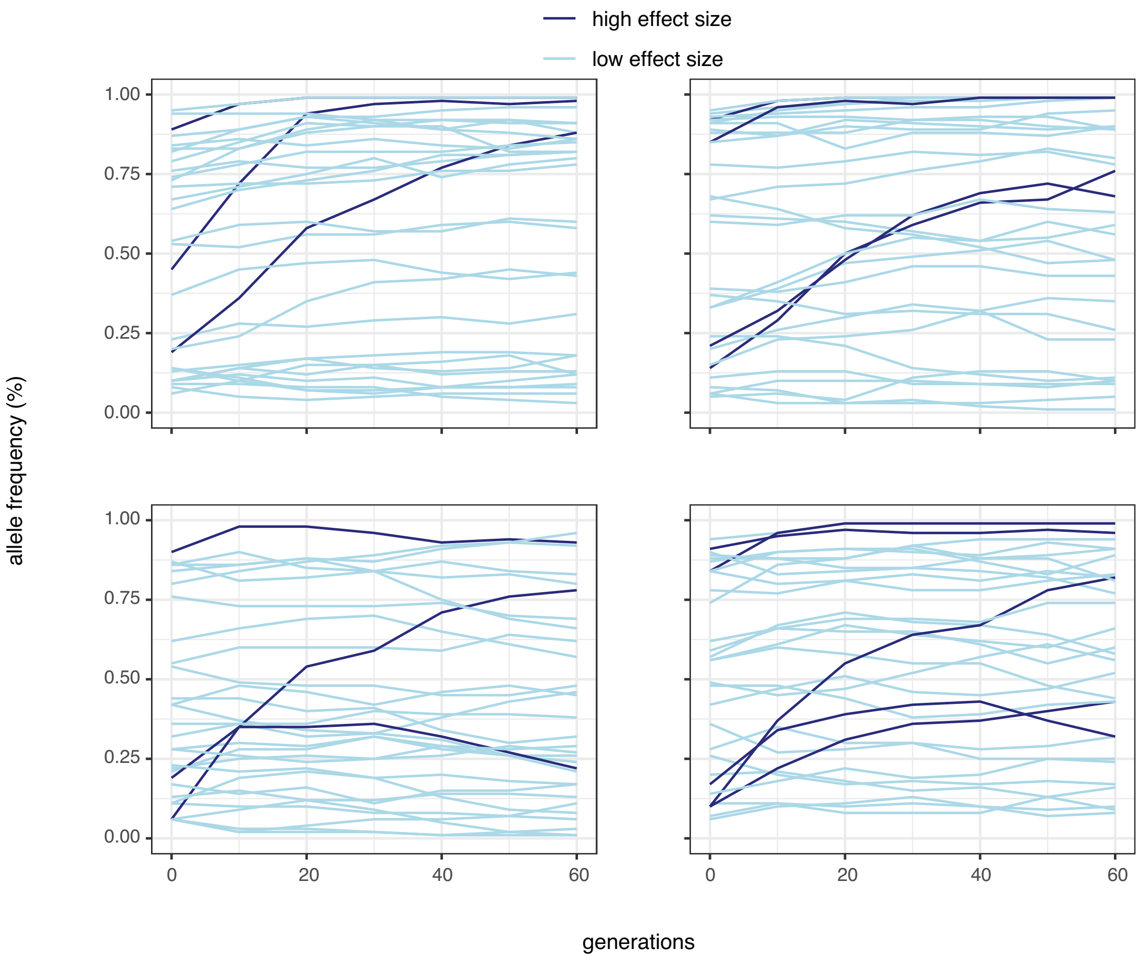

Fig. S6: Trajectories of the quantitative trait loci for stabilizing selection. Trajectories are shown for strong (dark blue; $e > 1$) and weak effect loci (light blue; $e \leq 1$). Four randomly drawn replicates are shown (out of 1000 simulated ones).

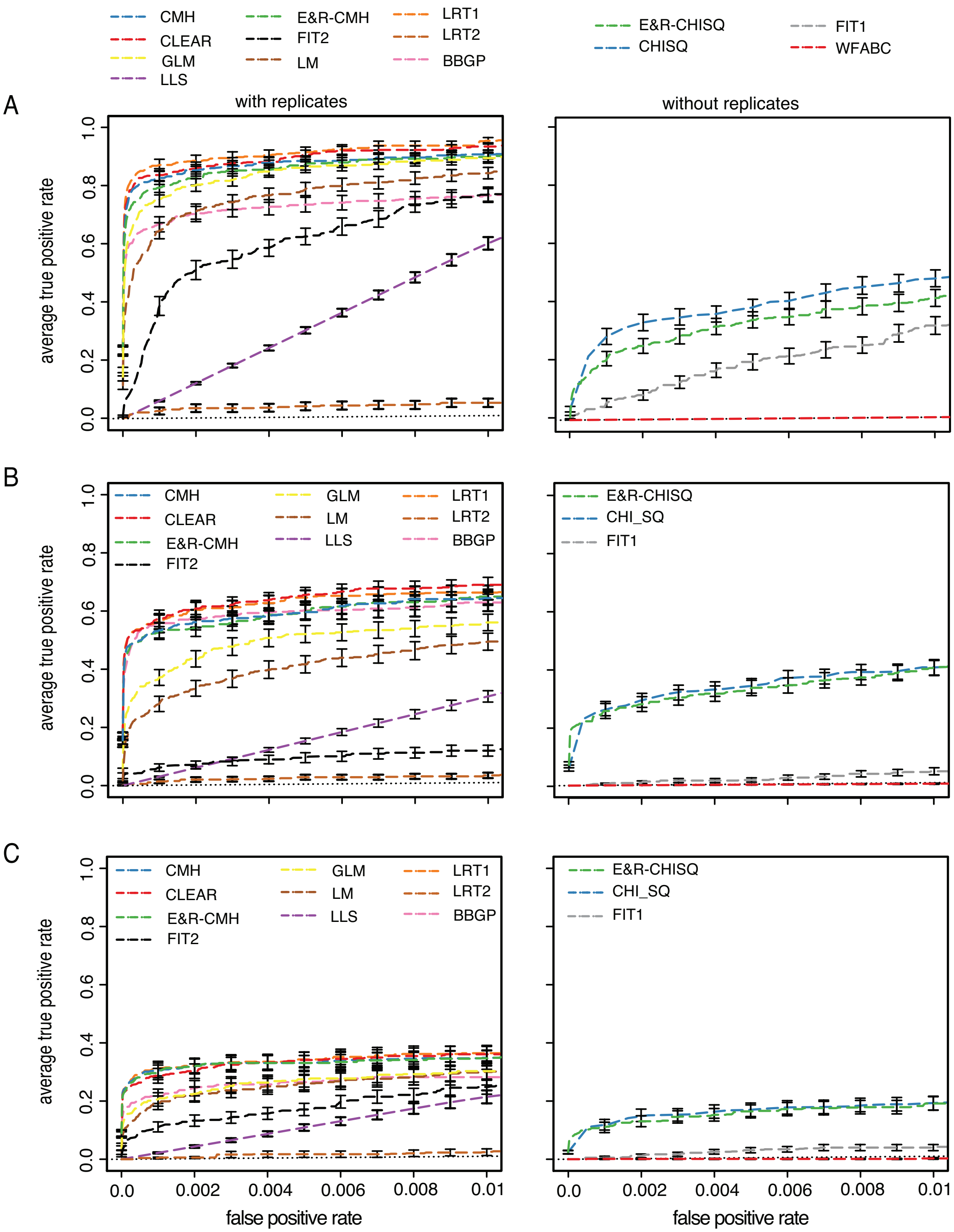

Fig. S7: Performance of the tools under three scenarios with a subset of the data (a 2Mb region of chromosome 2L). The performance of tools supporting replicates (left panels) and not supporting (right panels) replicates was analyzed separately. The performance of a random classifier is shown as reference (black doted line) A) selective sweeps B) truncating selection C) stabilizing selection

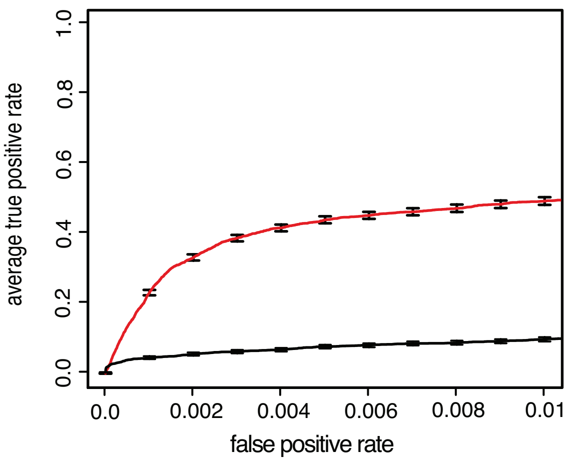

Fig. S8: Performance of FIT2 for truncating selection with (red) and without (black) a small Gaussian random number ($\mu = 0, \sigma = 0.00001$) added to the F60 allele frequencies.

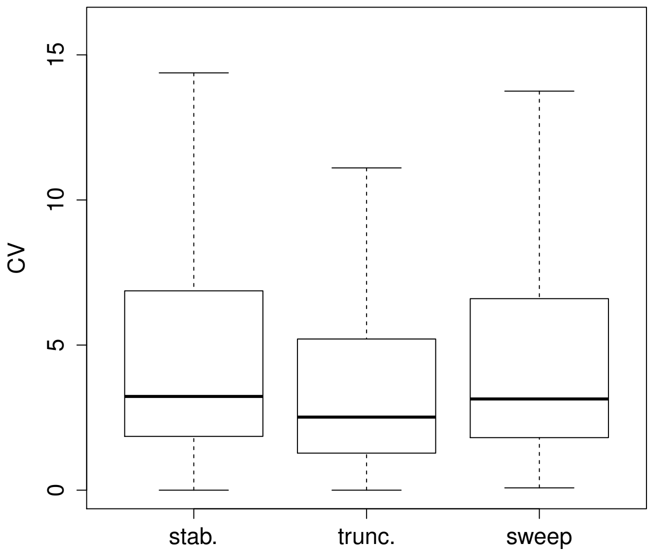

Fig. S9: Heterogeneity of the response to selection in the three scenarios. The boxplots display the coefficient of variation (CV) defined as $\frac{sd(dx_r)}{\hat{\delta}}$ for all targets of selection (3000; outliers not shown). The median CV per scenario is as follows: $CV_{stab.} = 3.230$, $CV_{trunc.} = 2.518$, $CV_{sweep} = 3.144$.

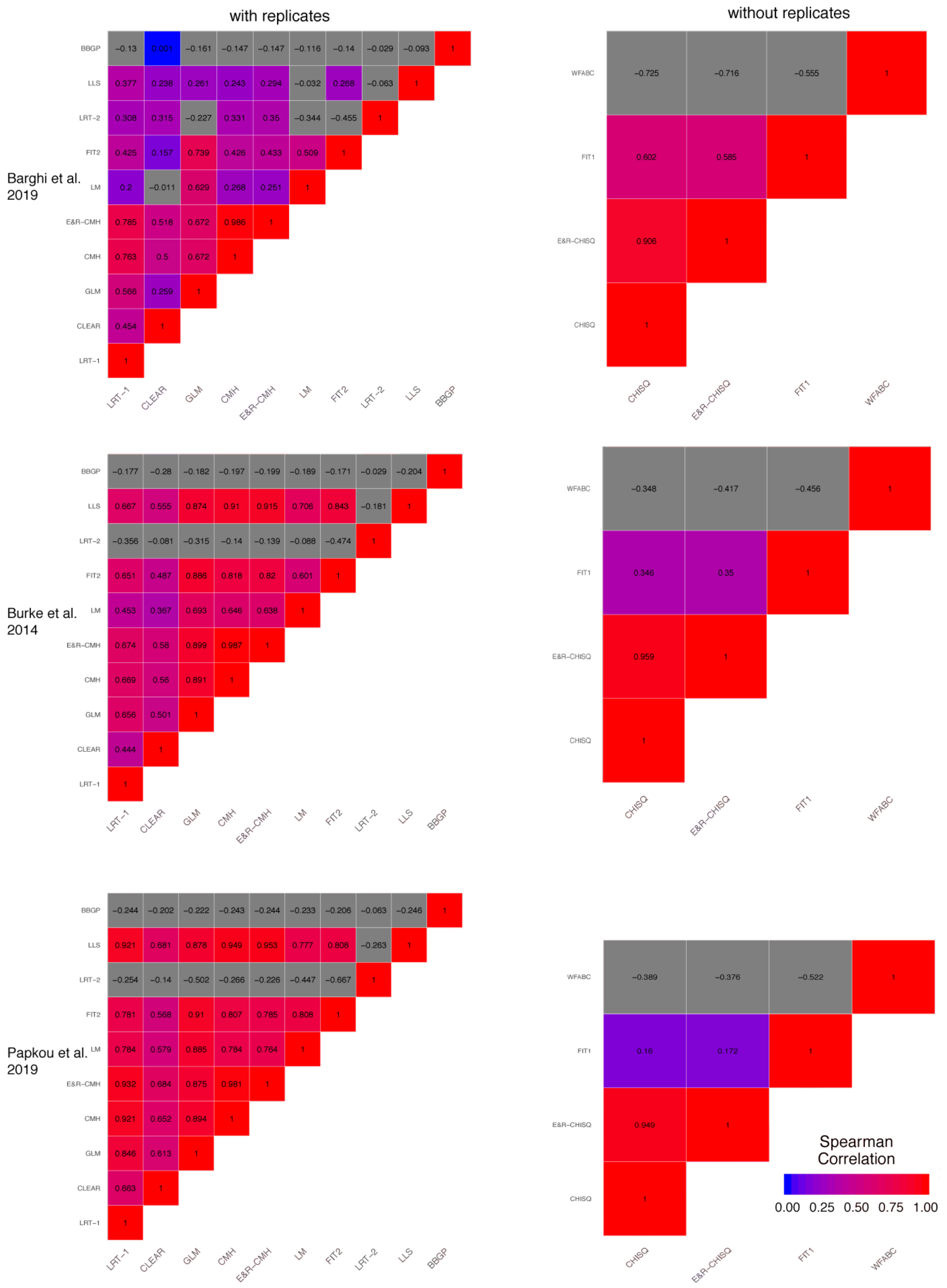

Fig. S10: Correlation of the test statistics for the data of Barghi et al. (2019), Papkou et al. (2019) and Burke et al. (2014)

Supplementary tables

Table S1: Technical difficulties encountered with different tools. We were thus unable to evaluate the performance of these tools.

tool	difficulty
Malaspinas et al. (2012)	The tool could not be obtained because the author did not respond.
Stern et al. (2019)	Haplotype information is required for each time point
Ferrer-Admetlla et al. (2016)	We were not able to find parameters that yield estimates of s that differed from 0.
Steinrücken et al. (2014)	We were not able to obtain positive likelihoods in all scenarios.
Bollback et al. (2008)	Tool not available
Schraiber et al. (2016)	Requirements not fulfilled. The sample size needs to be small relative to the population size but in our simulations they are identical.
Terhorst et al. (2015)	Testing this tool exceeded our computing capacity: each replicate took around 480h and required a large amount of memory (several GBs) for a subset of the data (2MB region).
Sackman et al. (2019)	This method is almost identical to WFABC. The only difference is that this method allows for a skewed offspring distributions, which is however not expected for our simulated scenarios (Wright-Fisher simulations).

Table S2: Links to tools used in this study. In case a version is not available we provide the date of the download.

tool	version	link
CLEAR	01/24/2019	`https://github.com/airanmehr/CLEAR`
cmh	1201	`https://sourceforge.net/p/popoolation2/`
E&R-cmh	1.0.1	`https://github.com/MartaPelizzola/ACER`
LLS	0.3.5	`https://github.com/ThomasTaus/poolSeq`
LRT-1/2	03/27/2019	provided by the author (A. Feder)
GLM	03/20/2019	adapted from `https://github.com/RAWWiberg/ER_PoolSeq_Simulations`
LM	03/20/2019	adapted from `https://github.com/RAWWiberg/ER_PoolSeq_Simulations`
BBGP	0.1.4	`https://github.com/PROBIC/GPrank`
FIT1/2	03/27/2019	provided by the author (J. K. Kelly)
WFABC	1.1	`http://jjensenlab.org/software`
slattice	1.0	`https://github.com/mathii/slattice/`
χ^2	0.3.5	`https://github.com/ThomasTaus/poolSeq`
E&R-χ^2	1.0.1	`https://github.com/MartaPelizzola/ACER`

References

N. Barghi, V. Nolte, T. Taus, A. M. Jakšić, R. Tobler, F. Mallard, M. Dolezal, C. Schlötterer, R. Kofler, and K. A. Otte. Genetic redundancy fuels polygenic adaptation in *Drosophila*. *PLOS Biology*, 17(2):e3000128, 2019.

H. Bastide, A. Betancourt, V. Nolte, R. Tobler, P. Stöbe, A. Futschik, and C. Schlötterer. A Genome-Wide, Fine-Scale Map of Natural Pigmentation Variation in *Drosophila melanogaster*. *PLoS Genetics*, 9: e1003534, 2013.

J. P. Bollback, T. L. York, and R. Nielsen. Estimation of $2Nes$ From Temporal Allele Frequency Data. *Genetics*, 179, 2008.

M. K. Burke, G. Liti, and A. D. Long. Standing genetic variation drives repeatable experimental evolution in outcrossing populations of saccharomyces cerevisiae. *Molecular biology and evolution*, 31(12):3228–3239, 2014.

J. Comeron, R. Ratnappan, and S. Bailin. The Many Landscapes of Recombination in *Drosophila melanogaster*. *PLoS Genetics*, 8(10):e1002905, 2012.

A. Ferrer-Admetlla, C. Leuenberger, J. Jensen, and D. Wegmann. An approximate markov model for the wright–fisher diffusion and its application to time series data. *Genetics*, 203(2):831–846, 6 2016. ISSN 0016-6731. doi: 10.1534/genetics.115.184598.

A.-S. Malaspinas, O. Malaspinas, S. N. Evans, and M. Slatkin. Estimating allele age and selection coefficient from time-serial data. *Genetics*, 192(2):599–607, 2012.

A. Papkou, T. Guzella, W. Yang, S. Koepper, B. Pees, R. Schalkowski, M.-C. Barg, P. C. Rosenstiel, H. Teotónio, and H. Schulenburg. The genomic basis of red queen dynamics during rapid reciprocal host–pathogen coevolution. *Proceedings of the National Academy of Sciences*, 116(3):923–928, 2019.

A. M. Sackman, R. B. Harris, and J. D. Jensen. Inferring Demography and Selection in Organisms Characterized by Skewed Offspring Distributions. *Genetics*, 211(3):1019–1028, mar 2019. ISSN 0016-6731. doi: 10.1534/genetics.118.301684. URL `http://www.genetics.org/lookup/doi/10.1534/genetics.118.301684`.

J. G. Schraiber, S. N. Evans, and M. Slatkin. Bayesian Inference of Natural Selection from Allele Frequency Time Series. *Genetics*, 203, 2016.

M. Steinrücken, A. Bhaskar, and Y. S. Song. A novel spectral method for inferring general diploid selection from time series genetic data. *The annals of applied statistics*, 8(4):2203, 2014.

A. J. Stern, P. R. Wilton, and R. Nielsen. An approximate full-likelihood method for inferring selection and allele frequency trajectories from DNA sequence data. *bioRxiv*, 2019.

J. Terhorst, C. Schlötterer, and Y. S. Song. Multi-locus analysis of genomic time series data from experimental evolution. *PLoS genetics*, 11(4):e1005069, 2015.
