# Supplementary material for: Benchmarking software tools for detecting and quantifying selection in evolve and resequencing studies
Source: Genome Biol. 2019 Aug 15;20:169. doi: 10.1186/s13059-019-1770-8 (PMC6694636; doi:10.1186/s13059-019-1770-8)
Supplement: Supplementary file 2 — Review history. (DOCX 17 kb) [file 13059_2019_1770_MOESM2_ESM.docx]

Review History

**Comments to author and authors’ point-by-point responses to the reviewers’ comments:**

**Reviewer 1**

In many species, combined with the wide application of second-generation sequencing, population genetics studies have been used to identify gene loci involved in domestication selection, modern breeding selection, local adaptation and experimental evolution. In this benchmarking work, the authors compared 10 software tools that were designed for genomic analyses of experimental evolution studies. Although this benchmarking study is useful for the E&R analysis, I think the topic "E&R analysis" may be too narrow. Moreover, I have several concerns or suggestions for this benchmarking study:

*Response: We understand that the reviewer aims for the benchmarking of broadly applicable software tools. Nevertheless, the tools we used for benchmarking are specifically dedicated to E&R studies and cannot be applied to other data sets.*

*We do not agree that E&R is a too narrow application. In fact, it is a research area that is just starting to exploit its full potential. This is illustrated by the large number of citations for a review in Nature Reviews Genetics (PMID: 26347030) on E&R. Even more support for the popularity of the topic is the fact that >15 different test statistics for E&R were developed within recent years and we anticipate that this trend will continue.*

*Therefore we think that E&R is a thriving research field, that will substantially benefit from improved approaches for finding the targets of selection and our benchmarking study will be a landmark for the future E&R studies.*

1. Page 11, Lines 49-52, the E&R simulations for benchmarking study used the D. melanogaster for all simulation parameters. I suggest the authors should also consider other species with diverse polymorphism patterns, population structure recombination pattern, average gene size and mating systems.

*Response:We included novel analysis where we compare the performance of the tools with three different species: Drosophila simulans, Caenorhabditis elegans and Saccharomyces cerevisiae . These species have diverged several hundred million years ago (PMID: 19441060 ) and differ in polymorphism pattern (PMID:22984349), mating system and genome architecture (ISBN:0716799022,9780878934843).*

*We added a novel paragraph to the results and novel figures (Figure 4, supplementary figure 10). Overall, our analyses showed that the evaluated tools performed similarly across different data sets (see also reply to editor).*

2. As the authors mentioned in Introduction (Page 1, Line 57- Page 2, Line 10), there are many data sets for E&R

analysis publically available. Hence, besides the simulation data used for the benchmarking study, the real sequence

data from the E&R works (e.g., using the VCF files) should be used to compare the performances of the 10 software

tools.

*Response: We compared the performance of the tools with data from three different real E&R studies (see above)*

3. The E&R tools and the comparisons should be extended to the experiments in plants. Because many plants have the inbreeding habits, special recombination patterns or even polyploidy, the performances of the E&R tools may be different.

*We understand the interest of the reviewer to include plant data. Unfortunately, the tools evaluated in our benchmarking study build on experiments that were conducted over several generations. The plant experiments we identified typically cover only a single generation. We also scanned two recent reviews about E&R but did not find any reference to studies conducted with plants (PMID: 26347030 , 26758350 ).*

*We attribute this dearth of E&R in plants to the long generation time of plants (even for Arabidopsis this is 2 months, compared with 2 weeks in Drosophila , 3–4 days in Caenorhabditis elegans , and hours in microbes; PMID:22819306).*

*E&R studies with plants suffer from the additional limitations that inbreeding, polyploidy and low recombination rates (as mentioned by the reviewer) will make identification of the targets of selection very difficult (if not impossible).*

**Reviewer 2**

This reviewer find it a straightforward paper of benchmarking 15 methods that evaluate E & R (evolve and resequence) approach in evolution analyses and studies.

It deals with an important problem of how we can analyze experimental evolution data. The analyses and presentation of the results are all suitable. The results are clear. The interpretation of the results are also scientifically straightforward.

This is a very typical benchmark analyses paper and this reviewer find no concerns on all the aspects of the paper.

I recommend it to be accepted not because of any novel evaluation method, or novel finding, but simply it is a very timely test on a very important field of using experimental evolution.

Resequencing data can be extremely useful in the field of genomics and general biology.

More applications of experimental evolution in biology will be required.

The authors categorized three major selection domains and the comparison results reflect the expected.

*Response:* *We thank the reviewer for the positive feedback.*
